# Novel polymerase gamma (*POLG1*) gene mutation in the linker domain associated with parkinsonism

**DOI:** 10.1186/1471-2377-13-92

**Published:** 2013-07-18

**Authors:** Rachel Dolhun, Erin M Presant, Peter Hedera

**Affiliations:** 1 Department of Neurology, Vanderbilt University, 465 21st Avenue South, 6140 MRB III, Nashville, TN, USA

**Keywords:** Mitochondrial DNA polymerase gamma (*POLG1*), Parkinsonism, Progressive external ophthalmoplegia, Ataxia, Sensory neuropathy

## Abstract

**Background:**

Mutations in the *POLG1* gene have variable phenotypic presentations and a high degree of clinical suspicion is necessary for their recognition. Parkinsonism and ataxia are the most common movement disorders associated with *POLG1* mutations but no phenotype-genotype correlation has been established.

**Case presentation:**

We identified a male patient with progressive external ophthalmoplegia who also developed a progressive bradykinesia, rigidity and camptocormia in the third decade. Parkinsonism was partially responsive to dopaminegic replacement. His father and brother had reportedly similar clinical problems. Genetic analysis identified a novel mutation p.K512M in the *POLG1* gene.

**Conclusion:**

This report further expands the spectrum of *POLG1*-associated neurologic problems with the report of a novel mutation in the linker region of the gene, which are rarely associated with parkinsonism.

## Background

Mitochondrial DNA polymerase gamma (*POLG1*) is a nuclear-encoded protein critical for the synthesis, replication and repair of mitochondrial DNA (mtDNA). Abnormal function of this sole mtDNA polymerase leads to defective oxidative phosphorylation and ATP production. Impaired integrity of mtDNA with its depletion or deletion causes a broad spectrum of both non-neurological and neurological phenotypes [1]. Greater than one hundred pathogenic mutations in this enzyme have been found since it was first described [2]. These include, but are not limited to parkinsonism, chronic progressive external ophthalmoplegia (CPEO), cerebellar ataxia, sensory polyneuropathy, Alpers-Huttenlocher syndrome, which is characterized by progressive encephalopathy with seizures and hepatic failure, isolated myoclonic epilepsy or non-syndromic liver failure. [2-6] This wide variety of distinct and seemingly unrelated clinical problems with both autosomal dominant (AD) or recessive (AR) inheritance presents a significant challenge to clinicians and a high degree of clinical suspicion is typically crucial for the recognition of the underlying genetic mechanism. Here we report a novel mutation in *POLG1* located in the linker domain of this gene in a patient with parkinsonism and CPEO.

## Case presentation

Presented patient is a 79-year old Caucasian who developed first neurologic problems in his thirties with bilateral ptosis and double vision. The ophthalmologic examination showed a progressive ophthalmoplegia and he required several corrective eyelid surgeries to mitigate his ptosis. Shortly thereafter, he developed a shuffling gait and overall slowness of movements. He experienced a gradual progression of his rigidity, bradykinesia without any significant asymmetry, hypophonia and within 10–15 years he also developed camptocormia with subsequent postural instability (Additional file 1).

Parkinsonism was partially responsive to dopaminergic replacement with carbidopa/levodopa with the highest used dose of 1250 mg per day. Later, amantadine at the dose of 300 mg/day was added for his gait difficulties with reported improvement. Both medications were later reduced because of hallucinations. Dementia, treated with memantine and donepezil, was diagnosed at the age of 75 years.

Family history was positive for external ophthalmoplegia and Parkinson’s disease diagnosed in his father and brother. We could not personally examine his brother but available history indicated that he was diagnosed with Parkinson’s disease and reported a good response to 600 mg of levodopa per day.

We performed muscle biopsy because of suspicion for a mitochondrial disorder at the age of 70 years. Muscle biopsy obtained from the vastus lateralis muscle revealed nonspecific changes with some irregularities of cytochrome *c*-oxidase staining but ragged red fibers were not seen. These changes alone were not sufficient for the definite diagnosis of a mitochondrial myopathy. Repeat biopsy was considered with sampling of additional muscles but the patient declined. Brain MRI showed only mild scattered white matter abnormalities.

**Figure 1:**
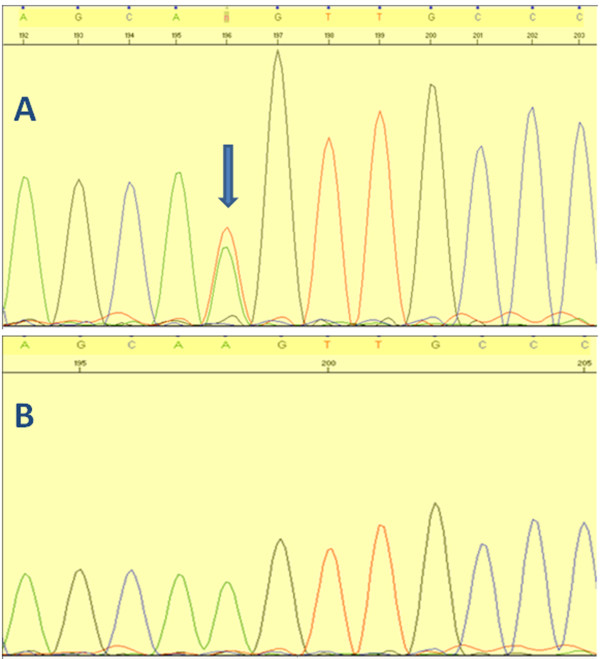
Panel A shows c.1535A>T mutation (arrow) and panel B normal sequence of the same segment.

**Figure 2:**
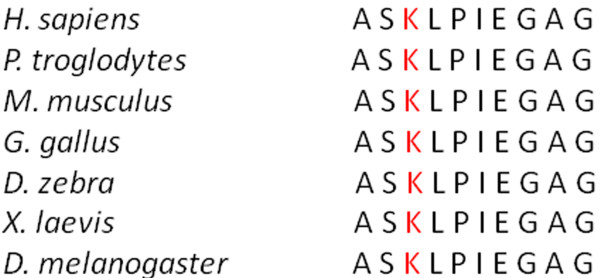
Alignment of the linker region of POLG1 protein with highly conserved amino acid residues among multiple species.

Sequencing of the *POLG1* gene was done by Sanger sequencing. DNA extraction from blood was carried out using standard procedures. All exons of the *POLG1* gene were amplified by PCR reaction with previously published intron-based primers [7]. Bidirectional Sanger sequencing was performed for each amplicon and detected single nucleotide polymorphisms (SNP) were compared with available public domain databases. We identified a novel heterozygous p.K512M mutation (c.1535A>T) (Figure 1). *In silico* analysis of this single nucleotide polymorphism using PolyPhen-2 software (http://genetics.bwh.harvard.edu/pph2) predicted that this change is possibly damaging with a score of 0.906 (sensitivity 0.82 and specificity 0.94). This is a highly conserved position in the linker region of the POLG1 protein (Figure 2) and sequencing of 100 normal, ethnically matched control individuals did not identify this sequence changes, further supporting its role as a disease-causing mutation.

Mutations in the *adenosine nucleotide translocator 1* (*ANT1*) and *twinkle* (*C10ORF2*) genes have also been associated with CPEO and additional neurologic problems, including parkinsonism [8,9]. We also sequenced all coding exons of these genes using the same approach as for *POLG1* analysis but no putative mutations were identified.

## Conclusions

The presented patient reflects a significant phenotypic variability associated with mutations in the *POLG1* gene. Parkinsonism is relatively rare phenotype caused by mutations in this gene. Previously suggested role of *POLG1* variants as a relatively common of idiopathic Parkinson disease (PD) has not been replicated, even though it is of interest because of evidence for mitochondrial dysfunction in PD [10]. However, the specific combination of CPEO and parkinsonism, which is partially responsive to dopaminergic therapy is occasionally seen in patients harboring *POLG1* mutations. [4,6,11-13] Most cases with this clinical phenotype carry heterozygous mutations in the polymerase domain of the *POLG1* gene and parkinsonism is a variable and inconsistent feature in these patients [4-6]. Mutations in the linker region of the *POLG1*gene are less common and limited data suggest that parkinsonism is more severe feature in these patients. The detected mutation p.K512M is the third known mutation in this functional domain causing CPEO and parkinsonism, together with the previously reported p.S511N and p.G517V mutations. [7,14] We could not confirm the segregation of p.K512M mutation with the disease within this small kindred because no additional family members were available for genetic analysis, but the lysine residue at the position 512 is highly conserved and sequencing of 200 normal chromosomes did not identify this variant in a normal population (Figure 2).

Similar phenotype of CPEO and parkinsonism can be also caused by mutations in the (*ANT1*) and mitochondrial DNA helicase *twinkle* genes [8,9]. These two genes were excluded as a potential cause of in our patient, which further strengthens the argument that the detected novel mutation in *POLG1* is indeed the disease-causing mutations. We did not detect any definite signs of mitochondrial skeletal muscle myopathy in spite of severe involvement of extraocular muscles. However, other *POLG1* patients with neurologic involvement and negative muscle biopsy were previously reported, suggesting that normal muscle biopsy does not exclude POLG1 mutations as a cause of multisystemic neurologic involvement [15].

Mutations in the *POLG1* gene have emerged as another great imitator of various movement disorders and a high index of suspicion is necessary for their correct identification. Cerebellar ataxia is one of the most genetically heterogeneous movement disorders and genotype-phenotype correlations are very unreliable and the rational algorithms for the genetic testing are only emerging. A combination of external ophthalmoplegia and/or polyneuropathy was caused by POLG1 mutations in 80% of patients who tested negatively for SCA1-3, and 6, and also did not have molecular evidence for Friedreich ataxia [7,16]. One of the most common mutations causing ataxia is p.A467T mutation that is also located in the linker domain but it causes neurologic disease only in a homozygous state. Furthermore, this mutation in a homozygous state is the most common cause of Alpers-Huttenlocher syndrome [17]. The reasons for this variability in phenotype-genotype correlation are unknown.

Parkinsonism associated with CPEO and cerebellar ataxia associated with upgaze palsy and neuropathy are easily recognizable syndromes where mutations in the *POLG1* gene should be considered. It is also important in furthering our understanding and research into the etiology of idiopathic Parkinson’s disease, particularly with regard to a possible contribution of mitochondrial dysfunction.

### Consent

Written informed consent was obtained from the patient for publication of this case report and to publish the videos. Additionally, informed consent was obtained for genetic analysis. Copies of the written consent are available for review by the Editor of this journal.

## Competing interests

The authors declare that they have no competing interests.

## Authors' contributions

RD performed clinical studies and helped to draft the manuscript. EMP performed clinical studies and helped to draft the manuscript. PH conceived of the study, its design, performed genetic analysis and helped to draft the manuscript. All authors read and approved the final manuscript.

## Pre-publication history

The pre-publication history for this paper can be accessed here:

http://www.biomedcentral.com/1471-2377/13/92/prepub

## Supplementary Material

Additional file 1 **The presented patient has bilateral ptosis and facial weakness.** He displays complete ophthalmoplegia when asked to move his eyes in any direction. Bilateral bradykinesia is evident. Gait is stooped and shuffling with slight anterocollis and truncal laterocollis. Click here for file
